# Physiologic response to sodium bicarbonate administration during pediatric in-hospital cardiac arrest

**DOI:** 10.3389/fmed.2026.1857577

**Published:** 2026-08-12

**Authors:** Jill L. Sorcher, Anne V. Grossestreuer, Nicole A. Duster, Monica E. Kleinman, Catherine E. Ross

**Affiliations:** 1 Division of Critical Care Medicine, Department of Anesthesiology and Critical Care Medicine, Children's Hospital of Philadelphia, Philadelphia, PA, United States; 2 Center for Resuscitation Science, Department of Emergency Medicine, Beth Israel Deaconess Medical Center, Boston, MA, United States; 3 Division of Critical Care Medicine, Department of Anesthesiology, Critical Care and Pain Medicine, Boston Children's Hospital, Boston, MA, United States; 4 Department of Anesthesiology, Perioperative and Pain Medicine, Brigham and Women's Hospital, Boston, MA, United States; 5 Division of Medical Critical Care, Department of Pediatrics, Boston Children's Hospital, Boston, MA, United States

**Keywords:** blood pressure, cardiac arrest, cardiopulmonary resuscitation, pediatric, sodium bicarbonate

## Abstract

**Aim:**

To explore the physiologic response to sodium bicarbonate (SB) administration during pediatric in-hospital cardiac arrest (p-IHCA) and its association with outcomes.

**Methods:**

Retrospective single-center study of patients < 18 years old who experienced IHCA with an arterial line in place and received intra-arrest SB from 2013 to 2023. To evaluate the hemodynamic response to SB in isolation, events in which vasopressors were administered the minute before, during or after the first dose of SB were excluded. The primary outcome was the change in diastolic blood pressure (DBP). Patients were classified as SB responders if DBP increased ≥5 mmHg. To assess for a potential enhancement on vasopressor efficacy, a secondary analysis was performed examining the difference-in-difference between the DBP response to epinephrine before and after SB administration.

**Results:**

Thirteen patients were included in the primary analysis, with a median DBP change of 4 mmHg (95% CI: −3, 11 after SB administration (*p* = 0.31). Of these, 6 (46%) patients were classified as SB responders. There were no significant differences in outcomes between SB responders and non-responders. Ten patients were included in the secondary analysis. There was no significant change in the difference-in-difference between the DBP response to epinephrine before and after SB administration [−2 mmHg (95% CI: −9, 7); *p* = 0.56].

**Conclusions:**

In this small cohort study, SB administration during p-IHCA was not associated with a significant change in DBP or an augmented DBP response to epinephrine. SB responder status was not associated with outcomes, though power to detect differences was low due to sample size.

## Introduction

Recent studies demonstrating a positive association between diastolic blood pressure (DBP) and improved outcomes from in-hospital cardiac arrest (IHCA) (1–4) support the widely-accepted physiologic premise that increasing DBP augments coronary perfusion pressure and thereby facilitates return of spontaneous circulation (ROSC) (1, 5–8). Based on this mounting evidence, international resuscitation guidelines endorse intra-arrest DBP monitoring for both adults and children (9, 10). While prior studies surrounding intra-arrest DBP optimization have largely focused on chest compression mechanics (11–13), understanding the physiologic response to pharmacological interventions may also be important (14).

Although DBP response to intra-arrest epinephrine has been described (8, 11–13, 15, 16), little is known about the physiologic response to other intra-arrest medications, such as sodium bicarbonate (SB). The 2025 American Heart Association Pediatric and Adult Advanced Life Support Guidelines recommend against the routine administration of SB during pediatric and adult cardiac arrest, except in cases of hyperkalemia, hypocalcemia and certain drug toxicities (17, 18). Despite these recommendations, SB is the second most commonly used drug in both adult and pediatric IHCA (19, 20). This apparent discrepancy between guideline recommendations and clinical practice is partially due to clinicians' beliefs that SB can mitigate the myocardial depression and diminished catecholamine responsiveness associated with intra-arrest acidosis, particularly during prolonged cardiac arrests (21–23). Therefore, we aimed to explore the physiologic response to intra-arrest SB. We hypothesized that DBP would increase following SB administration. Based on preclinical work showing that buffer therapy counteracts the negative effects of intra-arrest acidosis on myocardial contractility and response to catecholamines (22, 24), we also hypothesized that SB would potentiate the physiologic response to intra-arrest epinephrine, with a greater increase in DBP with *post-SB epinephrine* compared to *pre-SB epinephrine*.

## Methods

This retrospective cohort study was presented to the Institutional Review Board (IRB) at Boston Children's Hospital and was exempt from needing participant consent (IRB-P00044044). All study procedures were in accordance with the ethical standards of the responsible IRB and the Helsinki Declaration.

### Study population

The cohort was identified using quality improvement records of IHCA events at Boston Children's Hospital between January 1, 2013 and January 31, 2023. Patients < 18 years old who had an IHCA with chest compressions for ≥1 min, had an arterial line in place, and received at least one intra-arrest SB dose were included. Events occurring outside of the general or cardiac pediatric intensive care units (PICUs), events with incomplete data and non-index events were excluded. To isolate the effect of SB, events in which vasopressors were administered the minute before, during or after the first dose of SB were also excluded.

### Data collection and outcomes

Characteristics and outcomes were extracted from quality improvement records and manual chart review. Pre-arrest laboratory values within 2 h prior to IHCA were collected, if available.

**Figure 1:**
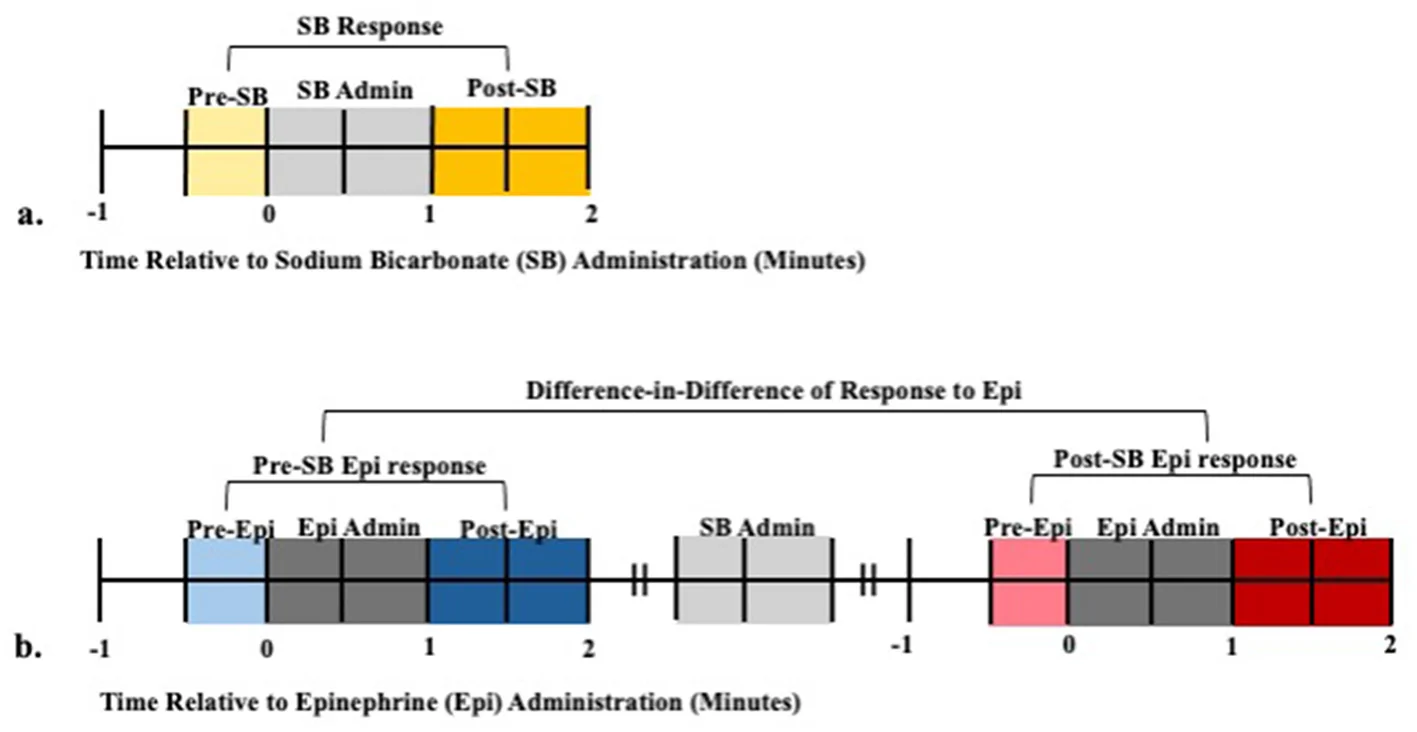
Timing of blood pressure sampling relative to intra-arrest drug administration. Time 0 is time of administration of drug of interest, sodium bicarbonate (SB) for primary analysis (**a**) and epinephrine for secondary analysis (**b**). The whole minute of CPR in which drug of interest was administered is not included in blood pressure analyses. The mean diastolic blood pressure (DBP) for the 30-sec epoch immediately preceding the minute before drug administration was used as the baseline, and the mean DBP from the whole minute following drug administration was considered the post-drug value.

For hemodynamic measurements, T3 (Etiometry, Inc, Boston, MA), a validated, FDA-cleared software application to collect monitor data which samples data every 5 sec, was used. The sampled invasive DBP measurements were averaged into 30-sec epochs. The epoch prior to the full minute of SB administration was used as the baseline DBP. The primary outcome was the change in DBP 1 min post-SB administration, defined as the average of the two epochs of ongoing CPR following the full minute in which SB was administered minus the baseline DBP (Figure 1a). Based on prior work surrounding intra-arrest epinephrine (15), patients were *a priori* classified as SB responders if DBP increased ≥5 mmHg.

To assess for enhancement on vasopressor efficacy, the secondary outcome was the difference-in-difference for the DBP response to epinephrine immediately before and after SB administration, defined as the change in DBP surrounding the post-SB epinephrine dose minus the change in DBP surrounding the pre-SB epinephrine dose (Figure 1b). For this analysis, the population was limited to patients who received at least one dose of epinephrine before and after SB and had complete DBP data 1 min before and after each epinephrine dose which did not overlap. Like the primary outcome, the epoch prior to the minute of epinephrine administration was used as the baseline DBP, and the post-epinephrine DBP was defined as the average of the 2 epochs after the full minute in which epinephrine was administered (Figure 1b). This cohort was *post hoc* classified as “post-SB epinephrine responders” if the difference-in-difference in DBP was ≥1mmHg.

### Statistical analysis

Descriptive statistics are presented as counts with proportions or medians with interquartile ranges (IQR) as appropriate.

For the primary analysis comparing the direct DBP response to SB administration within patients, a Wilcoxon signed-rank test was used to compare paired pre- and post-SB DBP values for the overall cohort. To estimate the magnitude of DBP change in response to SB, a Hodges-Lehman location shift estimator was calculated. This estimator, based on the Wilcoxon signed-rank statistic, is a robust, non-parametric effect estimate derived by calculating the median of all possible pairwise differences and is presented with a 95% confidence interval (CI) (25). A *post-hoc* power calculation for the primary analysis was performed which assumed a baseline median DBP of 34 mmHg (IQR: 28–46) based on prior work (15). For the 13 patient cohort, a minimum difference of 15 mmHg would be required for 80% power and an alpha of 0.05.

For the secondary analysis comparing the difference-in-difference in DBP response to epinephrine before and after SB between patients, Wilcoxon ranked sum test and Hodges-Lehman location shift estimator were used. This analysis was considered hypothesis generating given the small sample size.

Exploratory analyses were performed to assess the relationship between responder status, as defined in the primary and secondary analyses, and characteristics and outcomes. For these analyses, Fisher's exact tests and Wilcoxon ranked sum tests were used. Sensitivity analyses were conducted with threshold changes in DBP of 3 and 10 mmHg, respectively. Given the exploratory nature of this study, *p*-values were considered descriptive.

All analyses were performed using Stata 19.5 (College Station TX). A *p*-value of < 0.05 was considered statistically significant.

## Results

**Figure 2:**
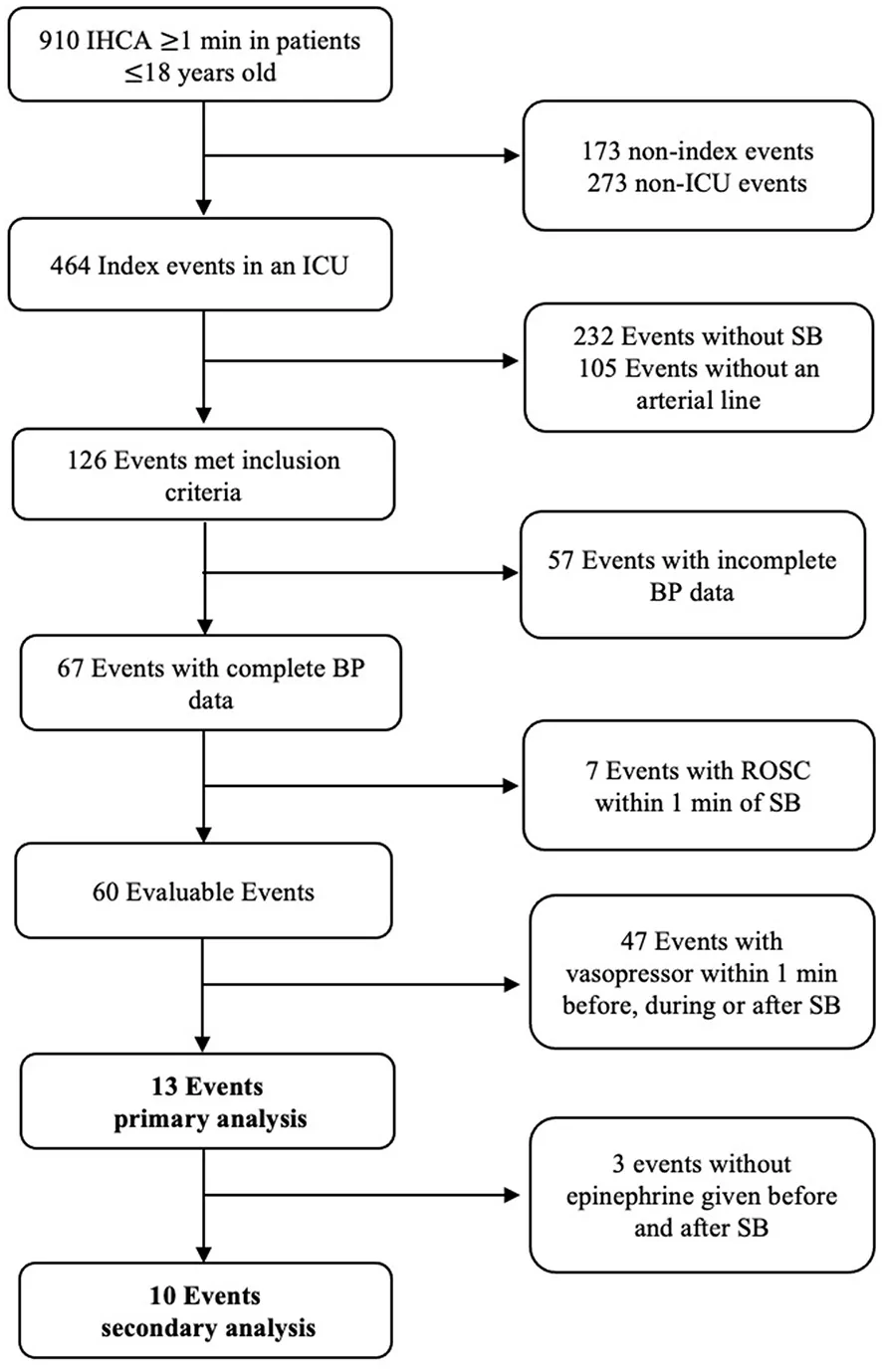
Consort diagram.

Thirteen events were included in the primary analysis. An additional sevan patients were excluded because ROSC was achieved within one 1 min of SB administration precluding evaluation of the primary outcome. The demographics and event characteristics of these patients were similar to the primary cohort (Figure 2, Supplementary Table S1).

The median age in the primary cohort was 0.4 (IQR: 0.1, 7.0) years (Table 1). Prior to arrest, 12 (92%) patients were receiving invasive mechanical ventilation, and 11 (85%) patients had vasoactive infusions. The median CPR duration was 20 min (IQR: 13, 32) and SB was given at a median 3 min (IQR: 3, 6). Ten patients had return of circulation, including 3 (23%) who achieved ROSC and 7 (54%) who required extracorporeal cardiopulmonary resuscitation. Four patients (31%) survived to hospital discharge.

**Table 1:** Baseline characteristics, event characteristics and outcomes according to SB responder status^*^.

Baseline characteristic	All (*n* = 13)	Responders (*n* = 6)	Non-responders (*n* = 7)	*P*-value
Age (years), median (IQR)	0.4 (0.1, 7.0)	0.2 (0.0, 7.0)	0.6 (0.2, 14)	NS
Female sex, *n* (%)	8 (62)	2 (33)	6 (86)	NS
Unit admitted, *n* (%)				NS
CICU	5 (38)	2 (33)	3 (43)	
PICU	8 (62)	4 (67)	4 (57)	
Illness category, *n* (%)				NS
Medical				
Cardiac	0 (0)	0 (0)	0 (0)	
Non-cardiac	3 (23)	1 (17)	2 (29)	
Surgical				
Cardiac	9 (69)	5 (83)	4 (57)	
Non-cardiac	1 (8)	0 (0)	1 (14)	
Pre-existing conditions, *n* (%)				
Acute CNS event	0 (0)	0 (0)	0 (0)	NS
Baseline depression in CNS function	0 (0)	0 (0)	0 (0)	NS
Acyanotic cardiac malformation	4 (31)	2 (33)	2 (29)	NS
Cyanotic cardiac malformation	8 (62)	5 (83)	3 (43)	NS
Metabolic abnormalities	2 (15)	1 (17)	1 (14)	NS
Metastatic or heme malignancy	3 (23)	1 (17)	2 (29)	NS
Pneumonia	1 (8)	1 (17)	0 (0)	NS
Respiratory insufficiency	3 (23)	0 (0)	3 (43)	NS
Renal insufficiency	1 (8)	0 (0)	1 (14)	NS
Sepsis	0 (0)	0 (0)	0 (0)	N/A
Interventions in place at time of event, *n* (%)				
Invasive mechanical ventilation	12 (92)	5 (83)	7 (100)	NS
Vasoactive infusions	11 (85)	6 (100)	5 (71)	NS
VIS, median (IQR)	29 (14, 39)	21 (11, 38)	34 (14, 76)	NS
Laboratory data (< 2 h prior to arrest), *n* (%)				
Hyperkalemia^$^	1 (25)	0 (0)	1 (33)	NS
pH acidosis^#^	0 (0)	0 (0)	0 (0)	N/A
15.6-7.2,-1.3498pt Bicarbonate acidosis^†^	1 (10)	0 (0)	1 (17)	NS
Event characteristics				
First sodium bicarb dose (meq/kg), median (IQR)	1.07 (0.96, 1.88)	1.38(0.96, 1.92)	1.02 (0.81, 1.88)	NS
Time to first SB dose, median (IQR)	3 (3, 6)	2.5 (2, 7)	4 (3, 6)	NS
Initial rhythm, *n* (%)				NS
Non-shockable	5 (38)	3 (50)	2 (29)	
Shockable	1 (8)	1 (17)	0 (0)	
Never pulseless	6 (46)	2 (33)	4 (57)	
Unknown pulseless	1 (8)	0 (0)	1 (14)	
Duration of CPR (minutes), median (IQR)	20 (13, 32)	25 (16, 32)	14 (12, 38)	NS
**Outcomes**				
ROSC, *n* (%)	3 (23)	2 (33)	1 (14)	NS
ROC, *n* (%)	10 (77)	6 (100)	4 (57)	NS
Survival to hospital discharge, *n* (%)	4 (31)	2 (33)	2 (29)	NS

^*^Defined as change in DBP ≥5 mmHg. ^$^Hyperkalemia was defined as a potassium > 6 mmol/L; 9 patients did not have lab data available. ^#^Acidosis was defined as pH < 7.0; 3 patients did not have lab data available. ^†^Acidosis was defined as bicarbonate < 15mmol/L; 3 patients did not have lab data available. IQR, interquartile range; NS, not significant; CICU, cardiac intensive care unit; PICU, pediatric intensive care unit; CNS, central nervous system; VIS, vasoactive-inotropic score; CPR, cardiopulmonary resuscitation; ROSC, return of spontaneous circulation; ROC, Any return of circulation including ROSC or extracorporeal cardiopulmonary resuscitation.

**Figure 3:**
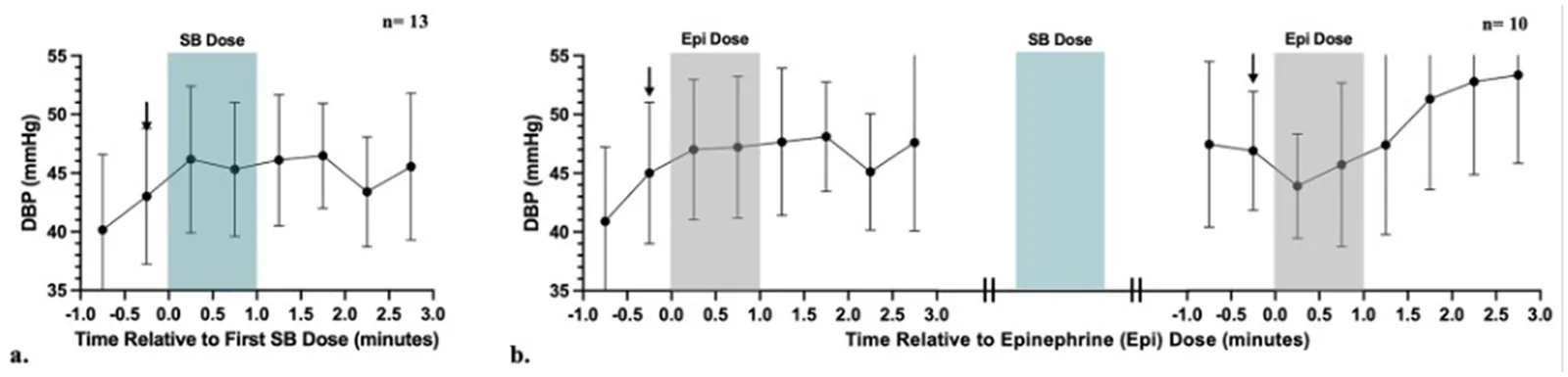
Temporal change in intra-arrest blood pressure relative to drug administration. Time 0 is time of administration of drug of interest, sodium bicarbonate (SB) for primary analysis (**a**) and epinephrine (epi) for secondary analysis (**b**). Arrows indicate baseline DBP measured during the 30-sec epoch immediately preceding drug administration. The whole minute of CPR in which the drug of interest was administered is shaded in blue (SB) or gray (epi) and was not included in blood pressure analyses. Each data point represents the mean DBP value for a 30-sec data epoch. Error bars indicate standard error of the mean for each data point.

For the primary outcome, the Hodges Lehman effect estimate for change in DBP with SB administration was 4 mmHg (IQR: −3, 11) (*p* = 0.31; Figure 3a, Table 2). The individual measurements for each patient are shown in Supplementary Table S2. Six (46%) patients were classified as SB responders. No significant differences in characteristics or outcomes between SB responders and non-responders were detected (Table 1, Supplementary Table S3). The sensitivity analyses using alternative cutoffs similarly showed no differences in outcomes (Supplementary Tables S4 and S5).

**Table 2:** Diastolic blood pressure response to sodium bicarbonate according to responder^*^ status.

Group	Measurement pairs, *n*	Pre-SB DBP	Post-SB DBP	Hodges-lehmann estimate of shift parameters (95% CI)	*P*-value
All	13	41 (31, 48)	42 (35, 59)	4 (−3, 11)	0.31
Responders	6	36 (20, 48)	52 (31, 59)	12 (7, 21)	0.03
Non-responders	7	41 (37, 71)	41 (35, 68)	−3 (−11, 0)	0.08

^*^Defined as change in DBP ≥5 mmHg. All values are mmHg and presented as median (IQR) unless otherwise specified. SB, sodium bicarbonate; DBP, diastolic blood pressure; CI, confidence interval; IQR, interquartile range.

After additional exclusions, 10 patients were included in the secondary analysis (Figure 2). The baseline and event characteristics for this cohort are shown in Table 3. The Hodges-Lehman effect estimate for the overall difference-in-difference following SB administration was −2 mmHg (95% CI: −9, 7; *p* = 0.56; Figure 3b, Supplementary Table S6). Four patients were classified as post-SB epinephrine responders. No significant differences between SB responders and non-responders were observed (Table 3).

**Table 3:** Baseline characteristics, event characteristics and outcomes according to post-SB epinephrine responder status, defined as a difference-in-difference in epinephrine response to SB ≥1 mmHg.

Baseline characteristic	All (*n* = 10)	Post-SB epinephrine responders (*n* = 4)	Post-SB epinephrine non-responders (*n* = 6)	*P*-value
Age (years), median (IQR)	0.5 (0.2, 14)	0.4 (0.1, 7)	4 (0.3, 14)	NS
Female sex, *n* (%)	7 (70)	3 (75)	4 (67)	NS
Unit admitted, *n* (%)				NS
CICU	3 (30)	2 (50)	1 (17)	
PICU	7 (70)	2 (50)	5 (83)	
Illness category, *n* (%)				NS
Medical				
Cardiac	0 (0)	0 (0)	0 (0)	
Non-cardiac	3 (30)	1 (25)	2 (33)	
Surgical				
Cardiac	6 (60)	3 (75)	3 (50)	
Non-cardiac	1 (10)	0 (0)	1 (17)	
Pre-existing conditions, *n* (%)				
Acute CNS event	0 (0)	0 (0)	0 (0)	N/A
Baseline depression in CNS function	0 (0)	0 (0)	0 (0)	N/A
Acyanotic cardiac malformation	2 (20)	0 (0)	2 (33)	NS
Cyanotic cardiac malformation	6 (60)	3 (75)	3 (50)	NS
Metabolic abnormalities	2 (20)	1 (25)	1 (17)	NS
Metastatic or heme malignancy	3 (30)	1 (25)	2 (33)	NS
Pneumonia	1 (10)	1 (25)	0 (0)	NS
Respiratory insufficiency	3 (30)	2 (50)	1 (17)	NS
Renal insufficiency	1 (10)	0 (0)	1 (17)	NS
Sepsis	0 (0)	0 (0)	0 (0)	N/A
Interventions in place at time of event, *n* (%)				
Invasive mechanical ventilation	9 (90)	4 (100)	5 (83)	NS
Vasoactive infusions	9 (90)	4 (100)	5 (83)	NS
VIS, median (IQR)	31 (14, 40)	37 (24, 40)	21 (11, 76)	NS
Laboratory data (< 2 h prior to arrest), *n* (%)				
Hyperkalemia,^$^	1 (25)	0 (0)	1 (33)	NS
pH acidosis,^#^	0 (0)	0 (0)	0 (0)	N/A
15.6-7.2,-1.3498pt Bicarbonate acidosis,^†^	1 (11)	0 (0)	1 (17)	NS
Event characteristics				
First sodium bicarb dose (meq/kg), median (IQR)	1.04 (0.96, 1.63)	1.35 (1.02, 1.75)	0.98 (0.81, 1.13)	NS
Time to first SB dose, median (IQR)	4 (3,7)	6.5 (4,12)	3 (3,6)	NS
Initial rhythm, *n* (%)				NS
Non-shockable	4 (40)	1 (25)	3 (50)	
Shockable	1 (10)	0 (0)	1 (17)	
Never pulseless	4 (40)	2 (50)	2 (33)	
Unknown pulseless	1 (10)	1 (25)	0 (0)	
Pre-SB epinephrine responder^*^	4 (40)	1 (25)	3 (50)	NS
Duration of CPR (min), median (IQR)	21 (14, 32)	17 (12, 26)	25 (16, 38)	NS
Event outcomes				
ROSC, *n* (%)	2 (20)	0 (0)	2 (33)	NS
ROC, *n* (%)	7 (70)	3 (75)	4 (67)	NS
Survival to hospital discharge, *n* (%)	2 (20)	1 (25)	1 (17)	NS

^*^Defined as change in DBP ≥5 mmHg to epinephrine pre-SB. ^$^Hyperkalemia was defined as a potassium >6 mmol/L; 6 patients did not have lab data available. ^#^Acidosis was defined as pH < 7.0; 1 patient did not have lab data available. ^†^Acidosis was defined as bicarbonate < 15mmol/L; 1 patient did not have lab data available. IQR, interquartile range; NS, not significant; CICU, cardiac intensive care unit; PICU, pediatric intensive care unit; CNS, central nervous system; VIS, vasoactive-inotropic score; CPR, cardiopulmonary resuscitation; ROSC, return of spontaneous circulation; ROC, Any return of circulation including ROSC or extracorporeal cardiopulmonary resuscitation.

## Discussion

We present the first description of the physiologic response to SB administration during cardiac arrest in humans. Contrary to our hypotheses, we failed to detect a significant change in DBP following intra-arrest SB or in the DBP response to intra-arrest epinephrine before and after SB; though power to detect differences was low due to small sample size.

Our findings complement prior work by Morgan et al. demonstrating a similarly modest DBP response to intra-arrest epinephrine with a median change of 4.4 mmHg (IQR: −1.9, 11.5), though this was not formally tested for significance. In their study, variation in DBP response across patients was observed, with only about half of patients meeting their definition for adequate response of ≥5 mmHg. Interestingly, we found a similar proportion of patients meeting this cutoff with SB despite no known direct vasopressor effects. Unlike Morgan et al., we were unable to detect differences in outcomes relative to responder status. However, it is notable that 7 additional events could not be evaluated due to ROSC occurring immediately after SB, suggesting beneficial physiological effects in these patients.

The physiologic effects of SB in critically ill humans are complex and may be altered by a variety of factors (26). For example, the variation in response to SB we observed may be explained by differences in underlying physiologies which may be more or less amenable to buffer therapy, including severity of acidosis, respiratory supports in place, adequacy of ventilation, duration and etiology of arrest and presence of pulmonary hypertension or myocardial dysfunction. Given its hyperosmolarity, SB may also increase venous return due to an osmolar effect, resulting in improved coronary perfusion pressure (21). It is possible that patients with hypovolemia may derive greater benefit from SB given its hyperosmolarity (27). Additionally, we also note that the median time to first SB dose in this cohort was only 3 min, suggesting that the profound acidosis associated with prolonged CPR may not yet have occurred; therefore, the effect of SB may have been less prominent. Future work should seek to identify subgroups of patients who may have greater DBP augmentation with intra-arrest SB.

Several limitations should be considered when interpreting this study. First, our small sample size resulted in limited power to detect changes in the DBP response to SB and associations with outcomes. Second, the eligibility criteria required for this study resulted in a severely ill cohort, as evidenced by the high rates of pre-arrest IMV and vasopressors, prolonged median arrest duration, and lower survival rate compared to the general pediatric IHCA population (28). This, in combination with being a single center study, may reduce external generalizability. Second, the physiologic response to SB administration may be impacted by several factors, including adequacy of ventilation, changes in chest compression quality metrics (depth, rate, recoil, compression fraction), changes in arrest rhythm, imprecision in detecting ROSC, etiology of arrest and arrest duration, which were unable to be controlled for in this small exploratory analysis. Third, neurologic outcome data was not available, limiting the granularity of long-term patient outcomes. Finally, given the challenges of IHCA documentation, inaccuracies in the timing of SB and epinephrine administration may have occurred.

## Conclusion

In this first clinical exploration of the physiologic response to intra-arrest SB, we did not detect a significant increase in DBP following SB, though power to detect differences was low due to small sample size. However, the magnitude and variation in DBP response to SB appeared similar to that of epinephrine in prior work.

## Data Availability

The raw data supporting the conclusions of this article will be made available by the authors, without undue reservation.
