# Supplementary material for: Physiologic response to sodium bicarbonate administration during pediatric in-hospital cardiac arrest
Source: Front Med (Lausanne). 2026 Aug 12;13:1857577. doi: 10.3389/fmed.2026.1857577 (PMC13523560; doi:10.3389/fmed.2026.1857577)
Supplement: Supplementary file 1 [file Supplementary_file_1.docx]

**Supplementary Material**

Contents

[Table S1. Demographics of the 7 patients who achieved ROSC within 1 minute of sodium bicarbonate administration.	2](#_Toc233868004)

[Table S2. Individual patient diastolic blood pressure response to sodium bicarbonate administration.	4](#_Toc233868005)

[Table S3. Vasoactive agents and dose according to sodium bicarbonate responder status defined as change in DBP ≥5 mmHg.	5](#_Toc233868006)

[Table S4: Diastolic blood pressure response to sodium bicarbonate according to responder status using alternative cutoffs for changes in DBP.	6](#_Toc233868007)

[Table S5: Outcomes according to responder status using alternative cutoffs for changes in DBP.	6](#_Toc233868008)

[Table S6: Diastolic blood pressure response to epinephrine before and after sodium bicarbonate.	7](#_Toc233868009)

[Table S6. STROBE Checklist	8](#_Toc233868010)

# **Table S1. Demographics of the 7 patients who achieved return of spontaneous circulation within 1 minute of sodium bicarbonate administration.**

| **Baseline Characteristic** | **All (n=7)** |
| --- | --- |
| Age (years), median (IQR) | 3 (0.4, 7) |
| Female sex, n (%) | 5 (71) |
| Unit Admitted, n (%) |  |
| CICU | 2 (29) |
| PICU | 5 (71) |
| Illness Category, n (%) |  |
| Medical |  |
| Cardiac | 0 (0) |
| Non-cardiac | 2 (29) |
| Surgical |  |
| Cardiac | 4 (57) |
| Non-cardiac | 1 (14) |
| Pre-existing Conditions, n (%) |  |
| Acute CNS Event | 0 (0) |
| Baseline depression in CNS function | 0 (0) |
| Acyanotic cardiac malformation | 1 (14) |
| Cyanotic cardiac malformation | 3 (43) |
| Metabolic abnormalities | 1 (14) |
| Metastatic or heme malignancy | 2 (29) |
| Pneumonia | 0 (0) |
| Respiratory insufficiency | 3 (43) |
| Renal insufficiency | 0 (0) |
| Sepsis | 0 (0) |
| Hypotension | 1 (14) |
| Interventions in Place at Time of Event, n (%) | |
| Invasive mechanical ventilation | 6 (86) |
| Vasoactive Infusions | 6 (86) |
| VIS, median (IQR) | 0 (0, 20) |
| Laboratory Data (<2 hours prior to arrest), n (%) | |
| Hyperkalemia* | 0 (0) |
| pH acidosis ^#^ | 0 (0) |
| Bicarbonate acidosis^†^ | 0 (0) |
| **Event Characteristics** | |
| First Sodium Bicarb Dose (meq/kg), median (IQR)^ | 1.1 (1.0, 2.0) |
| Time to first SB dose, median (IQR) | 4 (4, 5) |
| Initial Rhythm, n (%) | |
| Non-shockable | 4 (57) |
| Shockable | 1 (14) |
| Never pulseless | 0 (0) |
| Unknown pulseless | 2 (29) |
| Duration of CPR (minutes), median (IQR) | 5 (2, 5) |
| **Event Outcomes** | |
| ROSC, n (%) | 5 (71) |
| ROC, n (%) | 7 (100) |
| Survival to Hospital Discharge, n (%) | 5 (71) |

*Hyperkalemia was defined as a potassium > 6 mmol/L; 6 patients did not have lab data available

^#^Acidosis was defined as pH < 7.0; 4 patients did not have lab data available

^†^Acidosis was defined as bicarbonate < 15mmol/L; 6 patients did not have lab data available

^1 patient missing dosing data

Abbreviations: IQR, interquartile range; CICU, cardiac intensive care unit; PICU, pediatric intensive care unit; CNS, central nervous system; VIS, vasoactive-inotropic score; CPR, cardiopulmonary resuscitation; ROSC, return of spontaneous circulation; ROC, any return of circulation including ROSC or extracorporeal cardiopulmonary resuscitation

# **Table S2. Individual patient diastolic blood pressure response to sodium bicarbonate administration.**

| **Pre-SB DBP (mmHg)** | **Post-SB DBP (mmHg)** | **Change in DBP (mmHg)** |
| --- | --- | --- |
| 23 | 24 | 1 |
| 47 | 42 | -5 |
| 94 | 76 | -18 |
| 38 | 38 | 0 |
| 37 | 35 | -2 |
| 71 | 68 | -3 |
| 41 | 41 | 0 |
| 48 | 57 | 9 |
| 41 | 67 | 26 |
| 18 | 31 | 13 |
| 50 | 59 | 9 |
| 20 | 25 | 5 |
| 31 | 47 | 16 |

Abbreviations: SB, sodium bicarbonate; DBP, diastolic blood pressure

# **Table S3. Vasoactive agents and dose according to sodium bicarbonate responder status defined as change in DBP ≥5 mmHg.**

|  | **All** (n=13) | **Responders (n=6)** | **Non-Responders (n=7)** | ***P*-Value** |
| --- | --- | --- | --- | --- |
| Norepinephrine, n (%) | 11 (85) | 5 (83) | 6 (86) | NS |
| Dose | 0.01 (0.01, 0.1) | 0.06 (0.01, 0.10) | 0.01 (0.01, 0.35) | NS |
| Epinephrine, n (%) | 11 (85) | 5 (83) | 6 (86) | NS |
| Dose | 0.1 (0.05, 0.15) | 0.08 (0.05, 0.13) | 0.1 (0.01, 0.20) | NS |
| Milrinone, n (%) | 6 (46) | 2 (33) | 4 (57) | NS |
| Dose | 0 (0, 0.25) | 0 (0, 0.25) | 0.25 (0, 0.48) | NS |
| Vasopressin, n (%) | 4 (31) | 0 (0) | 4 (57) | NS |
| Dose, munit/kg/h | 0 (0, 3) | 0 (0, 0) | 3 (0, 35) | NS |
| Dopamine, n (%) | 11 (85) | 6 (100) | 5 (71) | NS |
| Dose | 6 (3, 7.5) | 5.5 (3, 7.5) | 7.5 (0, 10) | NS |

All values are mcg/kg/min and presented as median (IQR) unless otherwise specified

Patients not receiving vasoactive agent were assigned 0.

Abbreviations: DBP, diastolic blood pressure; NS, not significant; IQR, interquartile range

# **Table S4: Diastolic blood pressure response (DBP) to sodium bicarbonate according to responder status using alternative cutoffs for changes in DBP.**

| **Group** | **Measurement Pairs, n** | **Pre-SB-DBP** | **Post-SB-DBP** | **Hodges-Lehmann Estimate of Shift Parameters, (95% CI)** | ***P*-Value** |
| --- | --- | --- | --- | --- | --- |
| **Cutoff ≥3 mmHg** |  |  |  |  |  |
| All | 13 | 41 (31, 48) | 42 (35, 59) | 4 (-3, 11) | NS |
| Responders | 6 | 36 (20, 48) | 52 (31, 59) | 12 (7, 21) | NS |
| Non-responders | 7 | 41 (37, 71) | 41 (35, 68) | -3 (-11, 0) | NS |
| **Cutoff ≥10 mmHg** |  |  |  |  |  |
| All | 13 | 41 (31, 48) | 42 (35, 59) | 4 (-3, 11) | NS |
| Responders | 3 | 31 (18, 41) | 47 (31, 67) | N/A* | NS |
| Non-responders | 10 | 44 (37, 50) | 41 (35, 72) | 0 (-6, 5) | NS |

All values are mmHg and presented as median (IQR) unless otherwise specified

*95% CI could not be calculated due to the small number of observations

Abbreviations: SB, sodium bicarbonate; DBP, diastolic blood pressure; CI, confidence interval; IQR, interquartile range; NS, not significant

# **Table S5: Outcomes according to responder status using alternative cutoffs for changes in DBP.**

| **Outcomes** | **All (n=13)** | **Responders**  **(n=6)** | **Non-Responders**  **(n=7)** | ***P*-Value** |
| --- | --- | --- | --- | --- |
| **Cutoff ≥3 mmHg** |  |  |  |  |
| ROSC, n (%) | 3 (23) | 2 (33) | 1 (14) | NS |
| ROC, n (%) | 10 (77) | 6 (100) | 4 (57) | NS |
| Survival to Hospital Discharge, n (%) | 4 (31) | 2 (33) | 2 (29) | NS |
| **Cutoff ≥10 mmHg** |  |  |  |  |
| ROSC, n (%) | 3 (23) | 1 (17) | 2 (29) | NS |
| ROC, n (%) | 10 (77) | 3 (50) | 7 (100) | NS |
| Survival to Hospital Discharge, n (%) | 4 (31) | 1 (17) | 3 (43) | NS |

Abbreviations: DBP, diastolic blood pressure; ROSC, return of spontaneous circulation; ROC, any return of circulation including ROSC or extracorporeal cardiopulmonary resuscitation; NS, not significant

# **Table S6: Diastolic blood pressure response to epinephrine before and after sodium bicarbonate according to responder status*.**

| **Group** | **Measurement Pairs, n** | **Pre-SB  Epinephrine DBP Response (mmHg) (IQR)** | **Post-SB Epinephrine DBP Response (mmHg)** | **Hodges-Lehmann Estimate of Shift Parameters (mmHg) (95% CI)** | ***P*-Value** |
| --- | --- | --- | --- | --- | --- |
| All | 10 | -2 (-4, 3) | 1 (-2, 7) | -2 (-9, 7) | 0.557 |
| Responders | 4 | -4 (-6, -1) | 5 (1, 10) | N/A** | 0.125 |
| Non-responders | 6 | 2 (-3, 11) | -1 (-10, 3) | -6 (-18, -2) | 0.031 |

All values are mmHg and presented as median (IQR) unless otherwise specified

*Defined as difference-in-difference ≥ 1 mmHg

**95% CI could not be calculated due to the small number of observations

Abbreviations: SB: sodium bicarbonate; DBP: diastolic blood pressure; IQR, interquartile range

# **Table S6. STROBE Checklist**

|  | Item No. | Recommendation | Page  No. | Relevant text from manuscript |
| --- | --- | --- | --- | --- |
| **Title and abstract** | 1 | (*a*) Indicate the study’s design with a commonly used term in the title or the abstract | 2 | Retrospective cohort |
|  |  | (*b*) Provide in the abstract an informative and balanced summary of what was done and what was found | 2 |  |
| Introduction | | | |  |
| Background/rationale | 2 | Explain the scientific background and rationale for the investigation being reported | 3 |  |
| Objectives | 3 | State specific objectives, including any prespecified hypotheses | 3 | we aimed to explore the physiologic response to intra-arrest SB. We hypothesized that DBP would increase following SB administration. |
| Methods | | | |  |
| Study design | 4 | Present key elements of study design early in the paper | 3-5 |  |
| Setting | 5 | Describe the setting, locations, and relevant dates, including periods of recruitment, exposure, follow-up, and data collection | 4 | cohort was identified using  quality improvement records of IHCA events at Boston Children’s Hospital between January 1, 2013 and January 31, 2023. |
| Participants | 6 | (*a*) *Cohort study*—Give the eligibility criteria, and the sources and methods of selection of participants. Describe methods of follow-up  *Case-control study*—Give the eligibility criteria, and the sources and methods of case ascertainment and control selection. Give the rationale for the choice of cases and controls  *Cross-sectional study*—Give the eligibility criteria, and the sources and methods of selection of participants | 4 | Patients <18 years old who had an IHCA with chest compressions for ≥1 minute, had an arterial line in place, and received at least one intra-arrest SB dose were included. Events occurring outside of the general or cardiac pediatric intensive care units (PICUs), events with incomplete data and non-index events were excluded. |
|  |  | (*b*) *Cohort study*—For matched studies, give matching criteria and number of exposed and unexposed  *Case-control study*—For matched studies, give matching criteria and the number of controls per case | N/A |  |
| Variables | 7 | Clearly define all outcomes, exposures, predictors, potential confounders, and effect modifiers. Give diagnostic criteria, if applicable | 4-5 |  |
| Data sources/ measurement | 8* | For each variable of interest, give sources of data and details of methods of assessment (measurement). Describe comparability of assessment methods if there is more than one group | 4-5 |  |
| Bias | 9 | Describe any efforts to address potential sources of bias | 4-6 |  |
| Study size | 10 | Explain how the study size was arrived at | Figure 2 |  |

Continued on next page

| Quantitative variables | 11 | Explain how quantitative variables were handled in the analyses. If applicable, describe which groupings were chosen and why | N/A |  |
| --- | --- | --- | --- | --- |
| Statistical methods | 12 | (*a*) Describe all statistical methods, including those used to control for confounding | 5-6 |  |
|  |  | (*b*) Describe any methods used to examine subgroups and interactions | N/A |  |
|  |  | (*c*) Explain how missing data were addressed | 4-5 |  |
|  |  | (*d*) *Cohort study*—If applicable, explain how loss to follow-up was addressed  *Case-control study*—If applicable, explain how matching of cases and controls was addressed  *Cross-sectional study*—If applicable, describe analytical methods taking account of sampling strategy | N/A |  |
|  |  | (*e*) Describe any sensitivity analyses | N/A |  |
| Results | | | | |
| Participants | 13* | (a) Report numbers of individuals at each stage of study—eg numbers potentially eligible, examined for eligibility, confirmed eligible, included in the study, completing follow-up, and analysed | Figure 2 |  |
|  |  | (b) Give reasons for non-participation at each stage | Figure 2 |  |
|  |  | © Consider use of a flow diagram | Figure 2 |  |
| Descriptive data | 14* | (a) Give characteristics of study participants (eg demographic, clinical, social) and information on exposures and potential confounders | Tables 1 and 2 |  |
|  |  | (b) Indicate number of participants with missing data for each variable of interest | Tables 1 and 2 |  |
|  |  | (c) *Cohort study*—Summarise follow-up time (eg, average and total amount) | N/A |  |
| Outcome data | 15* | *Cohort study*—Report numbers of outcome events or summary measures over time | 6, Tables 1 and 2 |  |
|  |  | *Case-control study—*Report numbers in each exposure category, or summary measures of exposure | N/A |  |
|  |  | *Cross-sectional study—*Report numbers of outcome events or summary measures | N/A |  |
| Main results | 16 | (*a*) Give unadjusted estimates and, if applicable, confounder-adjusted estimates and their precision (eg, 95% confidence interval). Make clear which confounders were adjusted for and why they were included | 6, Figure 3, Tables S2 and S5 |  |
|  |  | (*b*) Report category boundaries when continuous variables were categorized | N/A |  |
|  |  | (*c*) If relevant, consider translating estimates of relative risk into absolute risk for a meaningful time period | N/A |  |

Continued on next page

| Other analyses | 17 | Report other analyses done—eg analyses of subgroups and interactions, and sensitivity analyses | 6 |  |
| --- | --- | --- | --- | --- |
| Discussion | | | | |
| Key results | 18 | Summarise key results with reference to study objectives | 7 |  |
| Limitations | 19 | Discuss limitations of the study, taking into account sources of potential bias or imprecision. Discuss both direction and magnitude of any potential bias | 7-8 | First, our small sample size resulted in limited power to detect changes in the DBP response to SB and associations with outcomes. Second, the eligibility criteria required for this study resulted in a severely ill cohort, as evidenced by the high rates of pre-arrest IMV and vasopressors, prolonged median arrest duration, and lower survival rate compared to the general pediatric IHCA population. This, in combination with being a single-center study, may reduce external generalizability. Finally, given the challenges of IHCA documentation, inaccuracies in the timing of SB and epinephrine administration may have occurred. |
| Interpretation | 20 | Give a cautious overall interpretation of results considering objectives, limitations, multiplicity of analyses, results from similar studies, and other relevant evidence | 8 | In this first clinical exploration of the physiologic response to intra-arrest SB, we did not detect a significant increase in DBP following SB, though power to detect differences was low due to small sample size. However, the magnitude and variation in DBP response to SB appeared similar to that of epinephrine in prior work. |
| Generalisability | 21 | Discuss the generalisability (external validity) of the study results | 7-8 | Our small sample size resulted in limited power to detect changes in the DBP response to SB and associations with outcomes. Second, the eligibility criteria required for this study resulted in a severely ill cohort, as evidenced by the high rates of pre-arrest IMV and vasopressors, prolonged median arrest duration, and lower survival rate compared to the general pediatric IHCA population. This, in combination with being a single center study, may reduce external generalizability. |
| Other information | |  | | |
| Funding | 22 | Give the source of funding and the role of the funders for the present study and, if applicable, for the original study on which the present article is based | 14 | The authors have no conflicts of interest or financial disclosures to report. |
